# Targeting the proper amyloid-beta neuronal toxins: a path forward for Alzheimer’s disease immunotherapeutics

**DOI:** 10.1186/alzrt272

**Published:** 2014-07-09

**Authors:** William F Goure, Grant A Krafft, Jasna Jerecic, Franz Hefti

**Affiliations:** 1 Acumen Pharmaceuticals, Inc., 4453 North First Street, #360, Livermore, CA 94551, USA

## Abstract

Levels of amyloid-beta monomer and deposited amyloid-beta in the Alzheimer’s disease brain are orders of magnitude greater than soluble amyloid-beta oligomer levels. Monomeric amyloid-beta has no known direct toxicity. Insoluble fibrillar amyloid-beta has been proposed to be an *in vivo* mechanism for removal of soluble amyloid-beta and exhibits relatively low toxicity. In contrast, soluble amyloid-beta oligomers are widely reported to be the most toxic amyloid-beta form, both causing acute synaptotoxicity and inducing neurodegenerative processes. None of the amyloid-beta immunotherapies currently in clinical development selectively target soluble amyloid-beta oligomers, and their lack of efficacy is not unexpected considering their selectivity for monomeric or fibrillar amyloid-beta (or both) rather than soluble amyloid-beta oligomers. Because they exhibit acute, memory-compromising synaptic toxicity and induce chronic neurodegenerative toxicity and because they exist at very low *in vivo* levels in the Alzheimer’s disease brain, soluble amyloid-beta oligomers constitute an optimal immunotherapeutic target that should be pursued more aggressively.

## Introduction

Alzheimer’s disease (AD) is the most common form of dementia, accounting for 60% to 80% of all dementias [1,2]. Worldwide, the prevalence of dementia was more than 35 million in 2010, and projections exceed 65 million by 2030 and 115 million by 2050 [1]. Significantly, no drugs that prevent or slow the progression of AD are currently approved. The development of effective AD therapeutics is clearly a tremendous medical challenge and should be one of society’s top medical priorities.

Despite the great need and significant societal and financial incentives, many pharmaceutical companies and investors have reduced investments in the search for new AD drugs, citing recent clinical failures of several high-profile experimental AD therapeutics and the high risks and costs of such development endeavors. The recent clinical failures also have intensified scrutiny of the ‘amyloid cascade hypothesis’, which spawned many of the recent experimental AD drugs targeting the amyloid-beta (Aβ) peptide. Nevertheless, the causal linkage between Aβ and AD remains strong and is supported by hundreds of studies over the past two decades [3-10]. (This is a representative sample of published reviews, and apologies are given to the authors of many excellent reviews that are not cited.)

Essentially all Aβ therapeutic approaches so far have targeted reducing the levels of Aβ monomer or Aβ deposits (or both) in the brain. However, today, the causal role of Aβ in AD is widely considered to involve soluble Aβ oligomers, and therapeutic strategies that selectively target soluble Aβ oligomers offer the potential to deliver rapid symptomatic benefit and long-term disease modification. This review describes the role of soluble Aβ oligomers within the amyloid hypothesis and discusses implications for current Aβ immunotherapies and new immunotherapies directed selectively toward soluble Aβ oligomers.

## The amyloid cascade hypothesis

The first suggestion of an ‘amyloid hypothesis’ to explain the pathology of AD was that of Wong and colleagues [11], who postulated that Aβ-derived cerebrovascular amyloid caused seepage of Aβ and other substances from plasma into the brain, leading to the formation of Aβ plaques and possibly neurodegeneration. This was revised into the more well-known amyloid cascade hypothesis that proposed that deposition of Aβ as neuritic plaques caused AD and led to neurofibrillary tangles, cell loss, vascular damage, and dementia [12]. The amyloid hypothesis linking Aβ to AD catalyzed much of AD and Aβ research over the past two decades, and key studies during that period led to important revisions of the hypothesis that highlighted the central role of soluble Aβ oligomers in synaptic dysfunction and loss [4,13-19].

The current understanding of the Aβ cascade is derived primarily from *in vitro* studies, the vast majority of which were conducted by using Aβ concentrations orders of magnitude greater than those found *in vivo*. The assembly of Aβ peptides to form soluble oligomers, protofibrils, and fibrils is well documented to be affected by the isoform of the starting peptide, how the peptide is treated prior to assembly, the nature of the buffer, pH, peptide concentration, assembly temperature and time, agitation, and presence of other peptides or biological materials [20-24]. Moreover, preparations of soluble Aβ species have been shown to change with time or upon dilution in different buffers, particularly in cell culture media [19]. Thus, caution must be taken in extrapolating the results and conclusions of *in vitro* studies to *in vivo* reality. Although precise mechanistic details remain to be elucidated, a multitude of studies by numerous researchers support the conclusion that monomeric Aβ peptides assemble to form soluble Aβ oligomers, which further aggregate to form fibrillar Aβ [17,25].

Three distinct pools of Aβ species exist: Aβ monomers, soluble Aβ oligomers, and insoluble fibrillar Aβ. Each of these pools encompasses an array of individual species. Thus, monomeric Aβ peptides encompass various isoforms, including Aβ(1-40), Aβ(1-42), and Aβ(1-43), as well as numerous N-terminal truncated isoforms. (For example, see the introductory paragraphs of Tekirian and colleagues [26].) Insoluble fibrillar Aβ aggregates are also known to be heterogeneous in structure and composed of various Aβ isoforms, both full-length as well as N-terminal and C-terminal truncated isoforms. (For example, see the introductory paragraphs of Roher and colleagues [27] and Thal and colleagues [28].)

Soluble Aβ oligomers are also heterogeneous and perhaps more ambiguous because of the different terminologies used by different researchers to describe them. (For an excellent review of soluble Aβ oligomers, see Benilova and colleagues [9].) Thus, soluble Aβ oligomer species reported by various researchers have been termed sodium dodecyl sulfate (SDS)-stable Aβ oligomers [29,30], low-n-oligomers [31-33], dimers [33-35], trimers [33,36-38], tetramers [37], paranuclei [38,39], dodecamers and Aβ*56 [37,40,41], amyloid-derived diffusible ligands (ADDLs) [42-44], Aβ oligomers [45], prefibrillar oligomers [46], Aβ globulomers [40,47-49], spherical oligomers [50], amylospheroids [51,52], protofibrils [20,53,54], and annular protofibrils [55,56]. Most of these terminologies refer to a mixture of metastable, soluble Aβ oligomer species in equilibrium rather than a discrete, stable species. In many cases, there is similarity in the species comprising the different preparations. In this review, we will use the terminology soluble Aβ oligomers to describe Aβ species composed of more than one Aβ peptide that remain in solution following centrifugation. Aβ fibrils or fibrillar Aβ will be used as a general description of insoluble Aβ plaques and vascular amyloid. (The term Aβ plaques rather than amyloid plaques will be used to more precisely describe amyloid plaques comprising primarily Aβ peptides versus those comprising primarily non-Aβ peptides.)

It has been established that the levels of Aβ monomer and deposited fibrillar Aβ in the AD brain are orders of magnitude greater than soluble Aβ oligomer levels [57-65]. However, more than three decades of intense investigation has not provided a precise understanding of the extent of interconversion among the various Aβ species.

Aβ dimers have been demonstrated to form at physiologically relevant concentrations of Aβ monomer *in vivo*[66,67]. Numerous *in vitro* studies have shown that soluble Aβ oligomers and protofibrils form under similar conditions and that both species can proceed to form larger fibrillar species [20,43,53,68-74]. More recent studies provide further support for the addition of soluble Aβ oligomers to protofibrillar and fibrillar assemblies and also provide data indicating that oligomerization can occur via a secondary nucleation mechanism caused by fibrillar Aβ [73,74], possibly suggesting a mechanistic linkage between fibrillar Aβ and soluble Aβ oligomers. However, the precise mechanism of *in vivo* fibril formation has not been fully established.

Aβ plaques are now generally considered to be a relatively benign species [19,72,75]; however, whether Aβ plaques are a sink or a source for toxic soluble Aβ oligomers is a subject of debate. Several studies with γ-secretase inhibitors [76-79] and two studies in transgenic mice that overexpress mutant amyloid precursor protein (APP) via a doxycycline-regulated promoter [80,81] show that subchronic or chronic suppression of Aβ production arrested Aβ plaque formation but did not result in observable clearance of existing plaques. In a related study [82], Aβ(1-42) immunization of Tg2576 mice prior to significant Aβ deposition, at an age with modest deposition or at an age with significant deposition, showed that immunization prevented additional Aβ deposition but did not result in significant clearance of pre-existing Aβ. These studies indicate that mature, dense-core Aβ plaques are not in significant equilibrium with soluble Aβ pools. Studies in transgenic mouse models of AD reporting decreased soluble Aβ oligomer levels and reduced cognitive deficits with increasing Aβ plaque levels also provide support for the concept that Aβ plaques may be a sink for soluble Aβ species [83,84].

However, in an elegant study in APP transgenic mice using a novel microdialysis technique, the half-life of low-molecular-weight Aβ species in hippocampal interstitial fluid following inhibition of Aβ production by a potent secretase inhibitor was doubled in 12- to 15-month-old mice with Aβ deposits compared with 3-month-old mice without Aβ deposits [85]. On the basis of these results, it was suggested that insoluble Aβ was in equilibrium with soluble Aβ in the interstitial fluid. In a related study using similar techniques, the temporal changes of low-molecular-weight Aβ species in interstitial fluid and Aβ levels in Tris-buffered saline (TBS), SDS, and formic acid extracts of brain tissues of 3-, 12-, and 24-month-old APP transgenic mice were reported [86]. A significant age-dependent decrease in low-molecular-weight Aβ in interstitial fluid and significant increases in Aβ species in SDS and formic acid extracts of brain tissues were found. Although Aβ increased approximately seven-fold from baseline in the TBS extract of brain tissues, the level of Aβ in TBS extracts was less than 2% of the total Aβ in SDS and formic acid extracts. These results indicated an age-dependent sequestration of Aβ as non-diffusible cell matrix and membrane-bound Aβ and deposited Aβ plaques. Acute γ-secretase inhibition of Aβ production in plaque-free and plaque-rich mice suggested that Aβ(1-42) in the interstitial fluid of plaque-rich mice was derived primarily from Aβ(1-42) sequestered in brain parenchyma rather than from new biosynthesis. However, it was not possible to determine whether cell matrix and membrane-bound Aβ or Aβ plaques or both forms of Aβ were the source of Aβ(1-42) in the interstitial fluid of aged mice.

In a more recent study of the temporal changes of Aβ species in the interstitial fluid and brain tissues of APP transgenic mice, Takeda and colleagues [87], using a 1,000-kDa-molecular-weight cutoff microdialysis probe, reported the temporal changes in soluble Aβ oligomer levels. In that study, consistent with previous studies [85,86], a significant, age-dependent increase in Aβ levels in TBS and formic acid extracts of brain tissues was found, and TBS extractable Aβ was less than 1% of formic acid extractable Aβ. However, unlike previous studies using a 35-kDa-molecular-weight cutoff microdialysis probe [85,86], a significant, age-dependent increase in interstitial fluid Aβ levels was found by using the larger-pore-sized microdialysis probe. The majority of the Aβ in interstitial fluid was determined to be higher-molecular-weight soluble Aβ oligomers, which showed an age-dependent increase relative to lower-molecular-weight Aβ species. Temporal changes in soluble Aβ oligomer levels in interstitial fluid and TBS brain extracts showed a significant positive correlation with formic acid Aβ extract levels. Comparison of high- and low-molecular-weight interstitial fluid Aβ levels at baseline and following acute treatment with a γ-secretase inhibitor showed slower clearance of higher-molecular-weight Aβ oligomers compared with low-molecular-weight Aβ species.

Narayan and colleagues [88] have recently used single-molecule imaging techniques to investigate interactions between Aβ peptides and hippocampal cell membranes and reported results indicating that Aβ oligomers preferentially interact with membranes compared with Aβ monomer, thereby providing support for the results observed in the microdialysis studies. Thus, this study, coupled with those of the temporal changes of Aβ species in APP transgenic mice [85-87], does not resolve the controversy regarding the sink/source relationship between fibrillar and soluble Aβ species.

The sink/source relationship between soluble and insoluble Aβ pools is complex, not fully understood, and subject to ongoing debate. Two different forms of Aβ plaques are present in the AD brain: vascular amyloid plaques that are primarily composed of Aβ(1-40) and Aβ plaques that are primarily composed of Aβ(1-42) [89]. *In vitro* studies have shown that fibrillar Aβ(1-40) and Aβ(1-42) are in equilibrium with soluble Aβ [90,91] but that recycling of Aβ(1-40) fibrils is significantly faster that Aβ(1-42) fibrils [91]. Moreover, it has been reported that plaque deposition proceeds in two distinct kinetic phases: an initial, reversible deposition phase followed by a time-dependent irreversible deposition phase [92]. Furthermore, a recent study of the rates of formation of Aβ oligomers and fibrils provided evidence that the formation of soluble Aβ oligomers from monomeric Aβ is catalyzed by fibrillar Aβ [73]. This study not only indicated a mechanistic linkage between soluble Aβ oligomers and fibrillar Aβ but also provided evidence showing that fibrillar Aβ can be a ‘source’ of soluble Aβ via catalyzed oligomerization of Aβ monomer rather than via a disaggregation process. This study also provided an alternative explanation for the study by Koffie and colleagues [93], who reported a halo of soluble Aβ oligomers surrounding Aβ plaques and proposed that Aβ plaques were a possible source of soluble Aβ oligomers.

Although precise details remain to be fully elucidated, collectively, over two decades of studies on the mechanisms of formation of oligomeric and fibrillar Aβ, the temporal distribution of Aβ species *in vitro* and *in vivo*, the age-dependent effect of Aβ immunization, and the effects of subchronic and chronic suppression of Aβ production upon *in vivo* Aβ plaque levels show a significant, age-dependent increase in brain levels of soluble Aβ oligomers and deposited fibrillar Aβ. Collectively, the studies suggest that mature, dense-core Aβ plaques are not in equilibrium to any significant extent with soluble pools of Aβ, but that Aβ sequestered in the cell matrix and membranes and immature plaques is in equilibrium with soluble Aβ pools, and that fibrillar Aβ catalyzes Aβ monomer oligomerization, giving rise to soluble Aβ oligomers and growth of Aβ plaques.

## Neuronal toxicity of amyloid-beta species

Monomeric Aβ, primarily the Aβ(1-40) and Aβ(1-42) peptides, is produced in various cell types throughout the body and reported to have trophic properties *in vitro*[94,95]. There are no reports suggesting that monomeric Aβ possesses any direct cellular toxicity at physiologically relevant concentrations. Insoluble fibrillar aggregates of Aβ, vascular amyloid and Aβ plaques, exhibit relatively low *in vitro* toxicity and have been proposed to be an *in vivo* mechanism for removal of the more toxic soluble Aβ species [83,96,97]. It was first suggested in 1995 that soluble Aβ species rather than fibrillar plaques could trigger neurotoxicity leading to AD [98], and in the subsequent decades, many studies have shown soluble Aβ oligomers to be the most toxic Aβ form, causing both acute synaptotoxicity and inducing neurodegenerative processes [5-10,99-102].

Low-picomolar levels of soluble Aβ oligomers have been reported to have trophic properties *in vitro*[103-105], suggesting that therapeutic targeting of soluble Aβ oligomers may need to modulate oligomer levels versus completely sequestering or preventing formation of soluble Aβ oligomers. However, the concentration of Aβ42 at which enhancement of long-term potentiation (LTP) was observed in these studies was one to two orders of magnitude greater than levels of soluble Aβ oligomers reported in the cerebrospinal fluid (CSF) of human patients with AD, and the concentration above which inhibition of LTP was observed was an order of magnitude greater than the total levels of soluble Aβ species reported in the CSF of human patients with AD [65]. Thus, the relevance of the reported *in vitro* trophic properties of soluble Aβ oligomers to *in vivo* conditions remains to be established.

Soluble Aβ oligomers bind with high affinity to synapses on a subset of hippocampal and cortical neurons [19,40,106-108], indicative of specific binding to discrete cell surface receptors. In rodent hippocampal slice preparations, synaptic binding leads to rapid inhibition of LTP [19,40,109], and injection of various soluble Aβ oligomer preparations directly into the rodent brain leads to reversible impairment of cognitive function [31,33,110]. This aberrant signaling also causes accumulated biochemical damage within neurons [100,111,112], such as hyperphosphorylation of tau [100,111-113], suggesting a linkage between Aβ and tau pathologies [114-116]. Soluble Aβ oligomers have been isolated from extracts of postmortem AD brain tissue and from transgenic AD animal models [37,52,117-119] and have been reported to be elevated in human AD brain relative to non-demented older patients [59,61,62,65,119-124]. Importantly, a recent study suggests a correlation between CSF levels of soluble Aβ oligomers and cognitive deficits in human patients with AD [61,65]. These findings support the view that soluble Aβ oligomers interfere acutely with normal synaptic functions and contribute significantly to the memory loss and cognitive dysfunction characteristic of AD.

## Structure and activity of soluble amyloid-beta oligomer species

There is substantial ongoing debate and research concerning the structure and activity of soluble Aβ species [5,7-9,33,41,52,72,99,125-127]. Various soluble Aβ oligomer species have been reported to display synaptic toxicity or induce cognitive deficits, including dimers [33,35,128,129], trimers [32], dodecamers [33,37,40], and larger soluble Aβ oligomers with molecular weights of 90 to 650 kDa (20 to 150 mers) [19,130,131]. Unfortunately, the different methodologies for the preparation, characterization, and evaluation of soluble Aβ oligomer species by various research groups impede a direct comparison of the results reported, and few studies have directly compared the toxicities of different soluble Aβ species while using the same techniques.

Two studies have reported the comparative toxicities of different soluble Aβ oligomer species by using LDH (lactate dehydrogenase release) and MTT (oxidoreductase activity) cell viability assays. Deshpande and colleagues [45] examined the relative toxicities of purified spherical Aβ(1-42) oligomers [50], ADDLs [132], and fibrils [50]. However, because solutions of soluble Aβ oligomers in the neurobasal medium used in this study have been shown to change with time [19], it is not possible to draw definitive structure-activity conclusions from the results of this study. In the second study, Ono and colleagues [133] reported relative toxicities of purified, cross-linked Aβ(1-40) dimers, trimers, and tetramers, which were shown to be relatively stable under the assay conditions. In an MTT assay, half maximal effective concentration values were 67.3, 41.6, 24.5, 20.5, and 57.6 μM, respectively, for monomer, dimer, trimer, tetramer, and fibrils. Comparable toxicity was obtained in an LDH assay. The micromolar concentrations of Aβ species used in this study were approximately six orders of magnitude greater than *in vivo* Aβ concentrations, and the relevance of cell culture MTT-type assays to the *in vivo* synaptotoxicity of Aβ species has been questioned [134]. Therefore, the results of the study by Ono and colleagues provide little understanding of the relative *in vivo* toxicities of soluble Aβ species.

More recently, cognitive effects *in vivo* were assessed in rats by using the alternating lever cyclic ratio assay following intracerebroventricular (ICV) injections of cell- and synthetically derived soluble Aβ oligomers [33]. Monomer and low-n-mer soluble Aβ oligomers derived from 7PA2 cells [29,135-137], trimer and a dodecameric species (Aβ*56) extracted from Tg2576 mouse brain [37], and synthetic soluble Aβ oligomers (ADDLs) [43] were compared in this study. Injection of conditioned media from 7PA2 cells caused significant cognitive deficits. Evaluation of size exclusion chromatography (SEC)-enriched dimer or trimer fractions of 7PA2 cell-conditioned media showed significant cognitive deficits following injection of dimer-enriched fractions but a non-significant effect upon injection of trimer-enriched fractions. SEC-purified monomer had no effect. Aβ monomer was the predominant Aβ species in unfractionated 7PA2 conditioned media, and the amounts of dimer and trimer injected in the dimer- and trimer-enriched fractions were considerably greater than the amounts injected in unfractionated conditioned media. However, cognitive effects following injection of unfractionated conditioned media were comparable to or exceeded the observed effects following treatment with dimer- and trimer-enriched fractions. Thus, there is an inherent ambiguity regarding the results reported for 7PA2-derived Aβ species that is difficult to explain. One possible explanation is that a higher-order soluble Aβ oligomer species contributes to the cognitive deficits observed upon injection of unfractionated 7PA2 conditioned media and is a more potent inhibitor of cognitive function than Aβ dimer or trimer species or both. Another possible explanation is that combinations of different soluble Aβ oligomer species, perhaps interacting differently with different neuronal receptors, have an additive or synergistic toxicological effect. Trimers extracted from aged Tg2576 mouse brain also failed to elicit significant cognitive deficits. However, consistent with other *in vivo* efficacy studies [37], a dodecameric soluble Aβ oligomer extracted from aged Tg2576 mouse brain (Aβ*56) caused significant cognitive deficits. Synthetic soluble Aβ oligomers (ADDLs) also caused cognitive deficits following ICV injection. The results of this study show that ICV injection of soluble Aβ oligomers from different sources causes cognitive deficits in wild-type rats and that these deficits are reversible. The results of the study show that soluble Aβ oligomer containing conditioned media of 7PA2 cells is more potent than solutions of Aβ*56 obtained from aged Tg2576 mouse brains or solutions of synthetically prepared soluble Aβ oligomers (ADDLs). However, because it is not possible to quantitatively characterize the exact nature or distribution of soluble Aβ species in these different preparations, it is not possible to draw definitive conclusions from this study regarding the structure-activity relationships between individual soluble Aβ oligomer species.

In a more recent study, Moreth and colleagues [19] prepared, characterized, and evaluated the hippocampal binding and effects on neurotransmission of spheroidal, protofibrillar, and fibrillar Aβ aggregates. Under the conditions used to test for hippocampal binding and neurotransmission, the different species where shown to be relatively unchanged. Monomeric and fibrillar Aβ did not bind to mature hippocampal neurons (DIV21) or effect neurotransmission at concentrations as high as 1 μM. In contrast, spheroidal and protofibrillar Aβ aggregates displayed punctate binding to mature hippocampal neurons and impaired neurotransmission with nanomolar potency. Significantly, the mode of impairment of neurotransmission was different for spheroidal Aβ aggregates, which impaired LTP at 30 nM, compared with protofibrillar aggregates, which impaired basal neurotransmission at 100 nM. Spheroidal Aβ aggregates had no effect on basal neurotransmission at concentrations as high as 100 nM. Although this study was unable to address the comparative neurotoxicity of discrete soluble Aβ oligomers, it did show that different forms of soluble Aβ oligomers can trigger distinct neuronal activities.

At this point, the exact structures of the toxicologically relevant soluble Aβ oligomer species have not been determined to the complete exclusion of other possible structures, and analytical tools do not exist to characterize the Aβ oligomers that form at concentrations in the AD brain [44,138]. The numerous studies reporting neuronal toxicity for different soluble Aβ oligomers support the conclusion that multiple soluble Aβ oligomer species exhibit neuronal toxicity, rather than a single, discrete toxic species. This suggestion of multiple toxic soluble Aβ oligomer species may also explain the plethora of reported neuronal receptors that mediate the effects of soluble Aβ oligomers (Table 1) [139-165], a discussion of which is well beyond the scope of this review. (See the perspective of Benilova and De Strooper [166] for a good introduction to this complex and controversial field of study.)

**Table 1:** Reported receptors that bind or mediate the toxicity of soluble amyloid-beta oligomers

**Reported receptor**		
Amylin receptor [139]	NMDA receptor [140-144]	Sortilin [145]
P53-Bax cell death pathway [146]	cAMP/PKA/CREB-signaling pathway [147,148]	Tau protein kinase 1/glycogen synthase kinase-3β [51]
c-Jun N-terminal kinase [149]	α-synuclein [150]	P/Q-type calcium currents [49]
Cyclin-dependent kinase 5 [149]	EphB2 [151]	Dynamin and RhoA [152]
mGluR, mGluR5, mGluR2 [108,142,149,153,154]	TNFα [155]	Cofilin [140]
p38 mitogen-activated protein kinase [149]	PrP^c^[154,156-159]	Calcineurin [160-162]
α 7-nicotinic receptors [105]	AMPA [160]	Brain-derived neurotrophic factor [131]
Human LilrB2 [163]	Insulin receptor [164]	Sigma 2 receptor [165]
STI1 [156]		

NMDA, N-methyl-D-aspartate; TNFα, tumor necrosis factor alpha.

Despite recognition that soluble Aβ oligomers are key structures causing AD memory malfunction and cognitive deficits, drug discovery efforts targeting these species have been hampered by perceived technical difficulties of generating physiologically relevant preparations of synthetic soluble Aβ oligomers and by differing terminologies and methodologies used by various researchers [167]. However, well-characterized and documented preparations of synthetic soluble Aβ oligomers have been reported by numerous researchers [19,40,43,95,100,107,108,111,168-170] that generate soluble Aβ oligomers with little or no detectable fibrillar Aβ species. With the availability of a number of well-documented preparations of different soluble Aβ oligomer species and tools for comparative characterization, it is hoped that additional side-by-side testing of various soluble Aβ oligomer preparations in different toxicity paradigms such as reversal of basal neurotransmission, LTP inhibition, changes in AMPA receptor trafficking, tau phosphorylation, and loss of dendritic spines [19,108,109,170] will be conducted and reported.

## Amyloid-beta immunotherapies in development

Successful immunotherapies developed for most diseases rely upon antibodies that possess high selectivity for a particular target antigen connected to the disease. However, all Aβ-directed immunotherapies currently in clinical development are based on non-selective antibodies that bind multiple Aβ species. Treatment with non-selective anti-Aβ antibodies that bind monomeric or oligomeric Aβ (or both) potentially could succeed in a prodromal treatment paradigm by reducing total brain Aβ to levels below the toxicological threshold. However, given the high concentrations of monomeric and fibrillar Aβ compared with soluble Aβ oligomers in the AD brain [57-65] and the low levels of antibodies that penetrate the brain from the periphery [171], it will be very challenging for non-selective anti-Aβ antibodies that bind monomeric or fibrillar Aβ (or both) to show efficacy when administered to patients with mild cognitive impairment or mild-to-moderate AD. In contrast, the acute synaptic toxicity and very low brain levels of soluble Aβ oligomers suggest that anti-Aβ antibodies with selective affinity for soluble Aβ oligomers could provide therapeutic benefit for patients with mild cognitive impairment or mild-to-moderate AD.

It might seem difficult to generate a monoclonal antibody that does not bind monomeric or fibrillar Aβ yet possesses high affinity for structurally heterogeneous soluble Aβ oligomers. However, monomeric, oligomeric, and fibrillar Aβ species have been reported to have distinct conformational characteristics [172-175]. Moreover, antibodies with selective affinity for soluble Aβ oligomers versus monomeric and fibrillar Aβ have been reported [9,176,177]. These several examples of antibodies suggest that therapeutically relevant soluble Aβ oligomer-directed antibodies are indeed feasible.

A number of recent publications have reviewed Aβ immunotherapies currently in clinical trials, with an emphasis on the efficacy of the drugs for treating AD [178-187], and the clinical results will not be presented in this review, with the exception of an analysis of the brain levels of solanezumab and bapineuzumab in recently reported clinical trials. Instead, this review will focus on a discussion of the comparative affinities of the Aβ antibodies in clinical development for monomeric, soluble oligomeric, and fibrillar Aβ species. Notably, none of the Aβ immunotherapies currently in clinical development selectively targets soluble Aβ oligomers, as discussed below and summarized in Table 2.

**Table 2:** Comparison of binding affinities of amyloid-beta (Aβ) immunotherapies in development for Aβ monomer, soluble Aβ oligomers, and fibrillar Aβ

	**Binding affinity**^**a**^		
**Aβ antibody**	**Aβ monomer**	**Aβ oligomers**	**Aβ plaque**
Solanezumab/266	+++	++	-
Bapineuzumab/3D6	++	+++	+++
Crenezumab	++	+++	++
Ponezumab/D-2H6	++	++	+++
BiiB037/NI-101.11	+	+++	+++
BAN2401/mAb158	-	+++	+++
Gantenerumab	+	++	+++
SAR228810/13C3	-	+++	+++

^a^Based on published patent applications or peer-reviewed scientific publications. +, low affinity binding; ++, moderate affinity binding; +++, high affinity binding; -, very low affinity or no binding.

### Solanezumab

Solanezumab is unique among Aβ antibodies currently in clinical development because it does not bind fibrillar Aβ. Solanezumab is a humanized, IgG1 monoclonal antibody derived from the murine monoclonal antibody, 266. 266 was selected for its high-affinity binding to soluble Aβ, although the exact nature of the soluble Aβ it binds was not reported. It has been suggested that 266 is a conformation-specific antibody that solely recognizes soluble Aβ and readily binds monomeric Aβ [188-190], without binding APP or the C-terminal APP cleavage product of α-secretase [189]. It has been reported that 266 selectively sequesters Aβ monomer and dimer in the periphery of 3-month-old APP transgenic mice [190], and definitive evidence for 266 binding of monomeric Aβ has been reported [191]. A study using crossed-linked soluble Aβ oligomers showed 266 could immunoprecipate Aβ monomer and low-molecular-weight dimer, trimer, and possibly tetramer [192]. A study involving a competitive ELISA showed that 266 had a seven-fold higher affinity for monomeric Aβ than synthetic soluble Aβ oligomers [193]. Several studies have shown that 266 does not bind Aβ plaques or vascular amyloid [188,190,194]. Thus, according to the available data, solanezumab and 266 bind monomeric and lower-molecular-weight soluble Aβ oligomer species, with a preference for monomeric Aβ, but do not bind fibrillar Aβ species.

Clinical trial pharmacokinetic data for brain levels of solanezumab have not been reported. However, target engagement data reported for the phase 2, multiple ascending-dose clinical trials (Table 3) [183] provide a possible understanding of the lack of efficacy of solanezumab in clinical trials [186]. The data for the 400 mg/month and 400 mg/week dose levels show approximately a 2.7-fold increase in solanezumab bound Aβ at the higher dose level (5,858 versus 15,825 pg/mL total Aβ40 and Aβ42) [183], showing that there was incomplete target engagement at the 400 mg/month dose level used in the pivotal phase 3 trials. Solanezumab binds Aβ monomer with higher affinity than soluble Aβ oligomers and does not bind or disaggregate fibrillar Aβ. Because incomplete target engagement occurred at the dose level used in the phase 3 trials, it is unlikely any solanezumab was available to bind soluble Aβ oligomers; thus, the lack of efficacy of solanezumab at the dose levels used in the phase 3 clinical trials is not unexpected.

**Table 3:** Mean cerebrospinal fluid levels of bound and unbound amyloid-beta (Aβ)40 and Aβ42 in Alzheimer’s disease patients treated with solanezumab

**Solanezumab dose**^**a**^	**Mean CSF Aβ40, pg/mL**^**b**^	**Mean CSF Aβ42, pg/mL**^**b**^
100 mg Q4W	625	0.0
100 mg QW	3,750	275
400 mg Q4W	5,625	233
400 mg QW	15,000	825

^a^Solanezumab phase 2 MAD data [183]. ^b^Mean pg/mL levels of total bound and unbound Aβ40 and Aβ42 in cerebrospinal fluid (CSF) of treated patients, derived from Figures 3A and 3B reported by Farlow and colleagues [183]. Q4W, monthly dosing; QW, weekly dosing.

### Bapineuzumab

Bapineuzumab is a humanized, IgG1 monoclonal antibody derived from the murine antibody, 3D6. Bapineuzumab and 3D6 are non-selective Aβ antibodies that bind with high affinity to monomeric Aβ [192], soluble Aβ oligomers [170], and fibrillar Aβ [195-197] but do not recognize APP or the product of β-secretase cleavage of APP [198]. In a competitive ELISA assay study, 3D6 was found to bind monomeric Aβ and synthetic soluble Aβ oligomers with similar affinity [193]. Babineuzumab/3D6 bind vascular amyloid and Aβ plaques in hAPP transgenic mice brains and human AD brains and clear vascular amyloid and Aβ plaques in hAPP transgenic mice brains [188,197,199-201]. Thus, bapineuzumab and 3D6 do not distinguish between the various Aβ species in the AD brain.

Mean CSF levels of bapineuzumab reported in the phase 2, multiple ascending-dose pharmacokinetic clinical studies were 4.9, 18.1, 27.2, and 44.7 ng/mL, respectively, at dose levels of 0.15, 0.5, 1.0, and 2 mg/kg (six infusions, 13 weeks apart) [202], which upon conversion to molar concentrations are 0.03, 0.12, 0.18, and 0.3 pmol/mL, respectively, based on a bapineuzumab molecular weight of 150 kDa. In the same studies, placebo-treated mean CSF levels of Aβ(1-42), Aβ(x-42), and Aβ(1-40) were 376.7, 537.9, and 6,705.4 pg/mL, respectively, which upon conversion to molar concentrations are 0.08, 0.12, and 1.55 pmol/mL, respectively (assuming the molecular weight of Aβ(x-42) is the same as that of Aβ(1-40)). The highest bapineuzumab dose level in phase 3 pivotal trials was 1 mg/kg (six infusions, once every 13 weeks) [187]. Thus, the total mean Aβ CSF levels were approximately an order of magnitude greater than mean levels of bapineuzumab. Because bapineuzumab binds monomeric Aβ with similar affinity to soluble Aβ oligomers and fibrillar Aβ and because Aβ soluble oligomer levels are orders of magnitude less than Aβ monomer and fibrils levels in the AD brain [58-65], it is clear that bapineuzumab was not dosed high enough in the pivotal phase 3 trials to effectively sequester soluble Aβ oligomers. Thus, the absence of efficacy of bapineuzumab in clinical trials is not unexpected.

### Crenezumab

Crenezumab (MABT5102A or MABT) is a humanized, IgG4 monoclonal anti-Aβ antibody that was engineered to reduce Fcγ receptor-mediated microglia activation and minimize adverse effects due to vasogenic edema and cerebral microhemorrhage [203,204]. Crenezumab binds Aβ monomer, soluble Aβ oligomers, and fibrillar Aβ with similar affinities and binds plaques in hAPP transgenic mice and human AD brain tissues [203,204]. Similar binding of crenezumab to human Aβ(1-40) and Aβ(1-42) peptides and mouse/rat Aβ(1-42) has also been reported [204]. These results indicate that crenezumab does not possess selectivity for different Aβ species [204]. Consistent with a lack of selectivity, crenezumab prevents Aβ aggregation and disaggregates pre-aggregated Aβ species. Thus, crenezumab is similar to bapineuzumab with respect to non-selective binding of monomeric, oligomeric, and fibrillar Aβ species.

### Ponezumab

Ponezumab is a humanized, modified IgG2 monoclonal antibody derived from the murine monoclonal antibody, 2H6. This progenitor murine antibody binds to monomeric Aβ(1-40) with high affinity but does not bind to Aβ(1-16), Aβ(1-28), Aβ(1-38), Aβ(1-42), or Aβ(1-43) [205]. Intracranial injection of 2H6 and its deglycosylated form into plaque-rich, 20-month-old Tg2576 mice followed by histological analysis of total Aβ load in the hippocampus and frontal cortex showed significant plaque reduction in both regions compared with control tissues [206], suggesting that these antibodies bind and disaggregate fibrillar amyloid. More recent studies show that ponezumab labels amyloid plaques in 14-month-old Tg2576 mouse and human AD brains [207], demonstrating binding to fibrillar Aβ. Binding of ponezumab to Tg2576 mouse brain plaque was comparable to the known high-affinity binding of the N-terminal Aβ antibody 6E10. Ponezumab was also reported to bind monomeric, oligomeric, and fibrillar forms of Aβ(1-40) but did not show appreciable binding to monomeric, oligomeric, or fibrillar forms of Aβ(1-42) [207]. Thus, although ponezumab displays selectivity for Aβ(1-40) versus other Aβ peptide isoforms, it is relatively non-selective for monomeric, soluble oligomeric, or fibrillar Aβ species.

### BiiB037

BiiB037 is a human IgG1 monoclonal antibody derived from a patient with AD by using reverse translational medicine methodology [208]. *In vitro* BiiB037 binds fibrillar Aβ(1-42) with high affinity but does not bind soluble Aβ(1-40) [209]. Although explicit details of the binding characteristics of BiiB037 have not been publicly disclosed, results reported by Dunstan and colleagues [209] and data in the patent covering BiiB037 [208] suggest that BiiB037 is very similar, or identical, to antibody NI-101.11, which binds with high affinity to Aβ plaques in human AD brain tissue samples [208]. Such binding is not blocked by monomeric Aβ(1-16) or Aβ(1-42), showing selective affinity for fibrillar Aβ versus Aβ monomers [208]. Comparison of binding affinity in an Aβ plate-based ELISA showed greater than 100-fold-higher affinity for fibrillar Aβ(1-42) than for monomeric Aβ(1-42) [208]. SEC studies showed that NI-101.11 bound to fluorescein isothiocyanate-labeled Aβ(1-42) in the early-eluting peak (non-retained, large Aβ species) but that little binding was associated with the retained, low-molecular-weight Aβ peak [208]. Many researchers have deployed SEC to separate higher-molecular-weight soluble Aβ oligomer species from low-molecular-weight Aβ species [20,44,130,155,210-212], and in all cases the higher-molecular-weight, early-eluting peak contains soluble Aβ oligomers or Aβ protofibrils or both. Together these results show that NI-101.11 binds soluble Aβ oligomers and protofibrils in addition to fibrillar Aβ. Consistent with this conclusion is the finding that NI-101.11 inhibits the formation of Aβ fibrils *in vitro*[208]. Thus, according to the available data, BiiB037/NI-101.11 binds soluble Aβ oligomers and fibrillar Aβ with high affinity and binds monomeric Aβ with low affinity.

### BAN2401

BAN2401 is a humanized, IgG2a monoclonal antibody derived from the murine precursor mAb158, which was raised against Aβ protofibrils generated from Aβ(1-42)-E22G, the arctic mutant of the Aβ peptide [210]. Although mAb158 is claimed to be a ‘highly protofibril-selective monoclonal antibody’ [213], published data show that it binds fibrillar Aβ with a high affinity similar to that of protofibrils in a dot-blot assay [210,214] and binds fibrillar deposits in transgenic APP-ArcSwe mice brain (see Figure S2 in [213]). In a more recent study, mAb158 was shown to bind soluble Aβ oligomer fractions in the molecular weight range of 80 to 2,000 kDa under conditions in which protofibrillar species were not detected [119]. In this study, mAb158 showed essentially no binding to low-molecular-weight Aβ oligomeric species in the molecular weight range of 5 to 80 kDa. A lack of binding to low-molecular-weight Aβ species - monomeric to tetrameric Aβ(1-40), Aβ(1-16), and Aβ(17-40) - was found in an earlier study [213]. Thus, mAb158 binds higher-molecular-weight Aβ aggregates, including soluble Aβ oligomers, protofibrils, and fibrillar Aβ. Consistent with the binding of mAb158 to fibrillar Aβ, intracranial injection of mAβ158 in a transgenic APP-Swe mouse model was reported to cause a significant reduction in plaque burden by 72 hours after injection [214]. In a subsequent study in a transgenic APP-ArcSwe mouse model [213], 4 months of weekly intraperitoneal injections of mAb158 to 9- to 10-month-old mice with modest plaque burden resulted in a significant reduction of Aβ protofibrils (74%) but no significant reduction in total soluble or insoluble Aβ levels in the cerebral cortex or hippocampus as determined by immunohistochemical analysis. The differences in effects of mAb158 on existing plaque burden in these two studies may be due to different administration routes (direct intracranial versus peripheral injections). As noted by Lord and colleagues [213], it may also be attributable to the APP-ArcSwe mouse model used in the more recent study, a model that exhibits more highly insoluble dense-core plaques, preventable only by early and continuous Aβ immunotherapy. Thus, mAb158/BAN2401 binds higher-molecular-weight soluble Aβ oligomers (referred to as protofibrils in the mAb158 literature) and fibrillar Aβ with high affinity and has low affinity for binding monomeric Aβ and lower-molecular-weight soluble Aβ oligomers.

### Gantenerumab

Gantenerumab is a humanized monoclonal antibody that was optimized to have high-affinity binding of fibrillar Aβ and shows high-affinity binding to diffuse and dense-core plaques in human AD brain and APP transgenic mouse brain tissues [215]. Gantenerumab also binds soluble Aβ oligomers with high affinity and, to a lesser extent, Aβ monomer. The reported binding constants (Kds) for gantenerumab binding to fibrillar Aβ, soluble Aβ oligomers, and Aβ monomer are 0.6, 1.2, and 17 nM, respectively [215]. The dissociation constants (kds) for gantenerumab-Aβ complexes were reported to be 2.8 × 10^-4^, 4.9 × 10^-4^, and 1.2 × 10^-2^, respectively, for fibrillar Aβ, Aβ oligomers, and Aβ monomer [215], suggesting a more rapid exchange of antibody-monomer complex compared with antibody-fibril or oligomer complexes. The selective affinity of gantenerumab for fibrillar Aβ and soluble Aβ oligomers suggests that it may be similar to BiiB013/NI-101.11 and BAN2401/mAb158. Consistent with its high-affinity binding to fibrillar Aβ, gantenerumab caused a significant, dose-dependent reduction in amyloid plaques in human AD brain tissue [215]. Immunohistochemical analysis of PS2APP transgenic mice brain tissue 3 days after intravenous injection of gantenerumab showed dose-dependent binding to amyloid plaques. Significantly, pharmacokinetic analysis in PS2APP transgenic mice following a single intravenous bolus dose showed sustained binding of gantenerumab to plaques in the brain for up to 9 weeks post-dosing, even though there was no detectable antibody in the plasma beyond 3 weeks post-dosing [215]. In a chronic treatment study in 5- to 6-month-old PS2APP transgenic mice, gantenerumab caused a significant reduction in pre-existing small plaques; however, there was an increase in larger plaques [215]. These results suggest that gantenerumab causes a redistribution of Aβ from small plaques to large plaques, possibly via disaggregation of smaller, less mature plaques. Gantenerumab-mediated disaggregation of existing plaques is consistent with the observations of vasogenic edema and microhemorrhage in human patients with AD treated with gantenerumab in clinical trials [216]. Thus, gantenerumab non-selectively binds soluble Aβ oligomers and fibrillar Aβ with similar high affinity in comparison with its lower-affinity binding of monomeric Aβ.

### SAR228810

SAR228810 is a humanized, monoclonal antibody derived from the murine monoclonal antibody 13C3, which was raised to Aβ protofibrils derived from Aβ(1-42) [217,218]. The humanized antibody was engineered into an IgG4 framework to reduce binding to Fcγ and C1q receptors and decrease risks of amyloid-related imaging abnormalities [219]. Although specific binding characteristics of SAR228810 have not been publicly disclosed, results reported by Pradier and colleagues [219] and data in the patent application claiming humanized variants of 13C3 [220] suggest that SAR228810 is very similar, or identical, to antibody LP09027 [220]. Antibody 13C3 and its humanized variants are reported to bind with higher affinity to SEC-separated protofibrillar Aβ(1-42) than to low-molecular-weight Aβ(1-42) [217,218,220]. As noted previously, many researchers have used SEC to separate higher-molecular-weight soluble Aβ oligomer species from low-molecular-weight Aβ species [20,44,130,155,210-212], and in all cases the higher-molecular-weight, early-eluting peak contains soluble Aβ oligomers or Aβ protofibrils or both. Moreover, as reported by Hepler and colleagues [44], the lower-molecular-weight, late-eluting peak contains predominately monomeric Aβ. Thus, these data show that 13C3 and its humanized variants selectively bind Aβ oligomers or protofibrils (or both) compared with monomeric Aβ. In competitive ELISA experiments, monomeric Aβ(1-28), Aβ(1-16), or Aβ(25-35) does not prevent binding of humanized 13C3 to plates coated with Aβ(1-42) [220]. Ravetch and Fukuyama [218] reported that 13C3 binds only to the higher-molecular-weight Aβ species contained in the supernatants from 7PA2 cells, in contrast to the non-selective Aβ antibody 4G8 that binds low-, medium-, and higher-molecular-weight Aβ species. Collectively, these data show the selectivity of 13C3 for higher-molecular-weight Aβ oligomers versus lower-molecular-weight Aβ oligomers and monomer. Antibody 13C3 was shown to bind fibrillar Aβ(1-42) and label Aβ deposits in human AD brain tissue samples [218]. Antibody LP09027 was shown to bind fibrillar Aβ *in vitro* and to label AB deposits in brain sections from APP-PS1 mice [220]. Thus, the reported data suggest that 13C3/SAR228810 is very similar to mAb158/BAN2401 and binds higher-molecular-weight soluble Aβ oligomers (referred to as protofibrils in the 13C3/SAR228810 literature) and fibrillar Aβ with high affinity and has low affinity for binding monomeric Aβ and lower-molecular-weight soluble Aβ oligomers.

## Conclusions

The lack of efficacy observed for Aβ immunotherapies in clinical development is not unexpected given their lack of selectivity for soluble Aβ oligomers compared with monomeric or fibrillar Aβ and given the tremendous quantities of monomeric or fibrillar Aβ in the AD brain relative to soluble Aβ oligomers. Aβ antibodies optimized to bind soluble Aβ oligomers selectively are much more likely to succeed in AD clinical trials and should be aggressively pursued.

The prediction that immunotherapies targeting soluble Aβ oligomers will elicit clinical benefit is supported by studies of human Aβ autoantibodies, of which only a subset appears to be disease-protective (in particular, the subset that preferentially recognizes Aβ oligomers) [221-223]. Thus, immunotherapeutics with high selectivity for soluble Aβ oligomers, which resemble these protective auto-antibodies, are expected to deliver a clinical advantage compared with the non-selective immunotherapies in clinical development.

Studies have demonstrated that antibodies with selective affinity for soluble Aβ oligomers can block soluble Aβ oligomer-mediated synaptotoxicity in cell cultures [108,224] and rapidly normalize memory deficits in transgenic AD mouse models [176]. These findings support the concept that antibodies with selective affinity for soluble Aβ oligomers may be a more effective therapeutic strategy than antibodies with high affinity for monomeric or fibrillar Aβ or both.

## Abbreviations

AD: Alzheimer’s disease; ADDL: Amyloid-derived diffusible ligand; APP: Amyloid precursor protein; Aβ: Amyloid-beta; CSF: Cerebrospinal fluid; ELISA: Enzyme-linked immunosorbent assay; ICV: Intracerebroventricular; LDH: Lactate dehydrogenase release; LTP: Long-term potentiation; SDS: Sodium dodecyl sulfate; SEC: Size exclusion chromatography; TBS: Tris-buffered saline.

## Competing interests

All authors are employees or paid consultants of Acumen Pharmaceuticals, Inc. and own stock or stock options of this company.

## Authors’ contributions

All authors read and approved the final manuscript.
